# Supplementary material for: Uptake and cardiac events of COVID-19 vaccinations among Canadian youth and young adults
Source: PLOS Glob Public Health. 2024 Jul 31;4(7):e0003363. doi: 10.1371/journal.pgph.0003363 (PMC11290663; doi:10.1371/journal.pgph.0003363)
Supplement: S1 Appendix — (DOCX) [file pgph.0003363.s001.docx]

**S1 Appendix.** Derived* Ontario’s COVID-19 Phase 2 vaccine guideline of at-risk health conditions.

|  | **Phase 2 Health Conditions** |
| --- | --- |
| **Solid organ transplant recipients** | Organ transplant recipients |
| **Hematopoietic stem cell transplant recipients** | Hematopoietic stem cell transplant recipients (bone marrow transplant) |
| **People with neurological diseases in which respiratory function may be compromised** | Neurological diseases that impact breathing (e.g., motor neuron disease, myasthenia gravis, multiple sclerosis) |
| **Hematological malignancy diagnosed < 1 year ago** | Hematological malignancy diagnosed within the last year |
| **Chronic kidney disease (with recent receipt of chronic dialysis)** | Kidney disease with estimated GFR <30 |
| **Current pregnancy** | Pregnancy |
| **High Risk Health Conditions** |  |
| **Other treatment causing immunosuppression** | Other treatments causing immunosuppression (for example, chemotherapy, immunity-weakening medications) |
| **Developmental disability** | Intellectual or developmental disabilities (for example, Down Syndrome) |
| **At risk** |  |
| **Immune deficiencies and autoimmune disorders** | Immune deficiencies and autoimmune disorders |
| **Stroke or cerebrovascular disease** | Stroke and cerebrovascular disease |
|  |  |
| **Dementia** | Dementia |
| **Liver disease** | Liver disease |
| **Chronic kidney disease** |  |
| **All other cancers** | All other cancers |
| **Respiratory diseases (COPD)** | Respiratory diseases, such as bronchitis, pneumonia, pleurisy |
| **Heart disease** | Heart disease |
| **Hypertension** | Hypertension with end organ damage |
| **Severe mental illness** | Diagnosed mental disorder |
| **Substance use disorder** | Substance use disorder |
| **Thalassemia** | Thalassemia |
| **Immunocompromising health conditions** | Immunocompromising health conditions Note: “Spleen” problems, such as “asplenia” and “Sickle cell disease” were combined with this characteristic. |
| **Pediatric conditions (12-17 years old only)** | |
| **Individuals with any pediatric complex chronic condition** |  |
| **Inflammatory bowel disease** |  |
| **Hemodynamically Significant Congenital heart disease (hs-CHD)** |  |
| **Bronchopulmonary dysplasia (BPD) or Congenital lung disease (CLD)** |  |
| **Cystic Fibrosis** |  |
| **Trisomy 21** |  |
| **Tracheostomy** |  |
| **Immunocompromising health conditions** |  |

This list and further information can be found at: https://news.ontario.ca/en/backgrounder/60570/populations-eligible-for-phase-two-covid-19-vaccination

* Asthma and Diabetes were removed from the list in the interest of our analyses
