# Supplementary material for: Uptake and cardiac events of COVID-19 vaccinations among Canadian youth and young adults
Source: PLOS Glob Public Health. 2024 Jul 31;4(7):e0003363. doi: 10.1371/journal.pgph.0003363 (PMC11290663; doi:10.1371/journal.pgph.0003363)
Supplement: S2 Appendix — (DOCX) [file pgph.0003363.s002.docx]

**S2 Appendix**. ICD and OHIP codes for atopic diseases and cardiac outcomes.

|  | **ICD-10-CA (DAD/NACRS)** | **DXCODE (OHIP)** |
| --- | --- | --- |
| ***Atopic Disease*** |  |  |
| Asthma | J45, J46 | 493 |
| Allergic rhinitis | J301-J304 | 477 |
| Eczema | L20 | 691.8 |
| ***Cardiac Outcomes*** |  |  |
| Myocarditis | I40-I41 | 391, 429 |
| Pericarditis | I30-I32 | 429 |

ICD-10-CA - International Classification of Diseases, Tenth Revision, Canadian Edition

DAD – Discharge Abstract Database

NACRS – National Ambulatory Care Reporting System
