# Supplementary material for: Uptake and cardiac events of COVID-19 vaccinations among Canadian youth and young adults
Source: PLOS Glob Public Health. 2024 Jul 31;4(7):e0003363. doi: 10.1371/journal.pgph.0003363 (PMC11290663; doi:10.1371/journal.pgph.0003363)
Supplement: S1 Table — (DOCX) [file pgph.0003363.s003.docx]

**S1 Table.** Likelihood of vaccination type from multivariable logistic regression models

|  | **Partial Vaccination vs Unvaccinated** | | | | **Full Vaccination vs Unvaccinated** | | | | **Boosted Vaccination vs Unvaccinated** | | | |
| --- | --- | --- | --- | --- | --- | --- | --- | --- | --- | --- | --- | --- |
| **Covariates** | **OR*** | **95% CI** | | **p-value** | **OR*** | **95% CI** | | **p-value** | **OR*** | **95% CI** | | **p-value** |
| *Population* |  |  |  |  |  |  |  |  |  |  |  |  |
| AAD^†^ | 1.19 | 1.17 | 1.22 | <.0001 | 1.34 | 1.33 | 1.35 | <.0001 | 1.39 | 1.39 | 1.40 | <.0001 |
| Diabetes | 1.19 | 1.04 | 1.35 | 0.0102 | 1.55 | 1.47 | 1.63 | <.0001 | 1.79 | 1.71 | 1.89 | <.0001 |
| Diabetes with AAD^†^ | 1.53 | 1.41 | 1.65 | <.0001 | 1.87 | 1.81 | 1.92 | <.0001 | 2.27 | 2.21 | 2.34 | <.0001 |
| General Population (reference) | 1.00 |  |  |  | 1.00 |  |  |  | 1.00 |  |  |  |
| *Sex* |  |  |  |  |  |  |  |  |  |  |  |  |
| Female | 0.89 | 0.88 | 0.90 | <.0001 | 0.92 | 0.92 | 0.92 | <.0001 | 1.22 | 1.21 | 1.23 | <.0001 |
| Male (reference) | 1.00 |  |  |  | 1.00 |  |  |  | 1.00 |  |  |  |
| *Age* |  |  |  |  |  |  |  |  |  |  |  |  |
| 12-17 | 1.76 | 1.73 | 1.79 | <.0001 | 2.94 | 2.92 | 2.95 | <.0001 | 1.71 | 1.70 | 1.73 | <.0001 |
| 18-35 (reference) | 1.00 |  |  |  | 1.00 |  |  |  | 1.00 |  |  |  |
| *Residence* |  |  |  |  |  |  |  |  |  |  |  |  |
| Urban | 1.00 | 0.98 | 1.03 | 0.9416 | 1.15 | 1.14 | 1.16 | <.0001 | 1.33 | 1.32 | 1.35 | <.0001 |
| Rural (reference) | 1.00 |  |  |  | 1.00 |  |  |  | 1.00 |  |  |  |
| *Income Quintile* |  |  |  |  |  |  |  |  |  |  |  |  |
| 1 (Lowest) | 1.21 | 1.17 | 1.26 | <.0001 | 1.27 | 1.25 | 1.29 | <.0001 | 0.91 | 0.90 | 0.92 | <.0001 |
| 2 | 1.16 | 1.12 | 1.19 | <.0001 | 1.22 | 1.21 | 1.23 | <.0001 | 0.96 | 0.95 | 0.97 | <.0001 |
| 3 | 1.13 | 1.10 | 1.16 | <.0001 | 1.19 | 1.18 | 1.20 | <.0001 | 0.94 | 0.93 | 0.95 | <.0001 |
| 4 | 1.12 | 1.09 | 1.15 | <.0001 | 1.21 | 1.20 | 1.22 | <.0001 | 1.02 | 1.01 | 1.03 | <.0001 |
| 5 (Highest) | 1.00 |  |  |  | 1.00 |  |  |  | 1.00 |  |  |  |
| *Recent Immigrant* |  |  |  |  |  |  |  |  |  |  |  |  |
| Yes | 2.04 | 1.99 | 2.09 | <.0001 | 2.61 | 2.59 | 2.63 | <.0001 | 2.34 | 2.32 | 2.37 | <.0001 |
| No (reference) | 1.00 |  |  |  | 1.00 |  |  |  | 1.00 |  |  |  |

* Also adjusted for instability, deprivation, dependency, and ethnic diversity quintiles and days followed
†AAD stands for asthma and allergic diseases
OR – Odds Ratio
95% CI – 95% confidence interval
