# Supplementary material for: Uptake and cardiac events of COVID-19 vaccinations among Canadian youth and young adults
Source: PLOS Glob Public Health. 2024 Jul 31;4(7):e0003363. doi: 10.1371/journal.pgph.0003363 (PMC11290663; doi:10.1371/journal.pgph.0003363)
Supplement: S2 Table — (DOCX) [file pgph.0003363.s004.docx]

**S2 Table.** Cardiac events by vaccine dose type and number

|  |  | **Cardiac Event** | | |  |
| --- | --- | --- | --- | --- | --- |
| **Dose Number** | **Vaccine Type** | **No** | **Yes** | **Total** | **p-value** |
| **Dose #1** | **Original** | **3646916** | **812-817** | **3647733** | **0.8894** |
|  |  | **99.98%** | **0.02%** |  |  |
|  | **Bivalent** | **5125-5131** | **<6** | **5131** |  |
|  |  | **>99.9%** | **<0.1%** |  |  |
|  | **Total** | **3652046** | **818** | **3652864** |  |
| **Dose #2** | **Original** | **3649807** | **1154** | **3650961** | **0.4379** |
|  |  | **99.97%** | **0.03%** |  |  |
|  | **Bivalent** | **1903** | **0** | **1903** |  |
|  |  | **100%** | **0%** |  |  |
|  | **Total** | **3651710** | **1154** | **3652864** |  |
| **Dose #3** | **Original** | **3604675** | **331** | **3605006** | **0.0892** |
|  |  | **99.99%** | **0.01%** |  |  |
|  | **Bivalent** | **47850** | **8** | **47858** |  |
|  |  | **99.98%** | **0.02%** |  |  |
|  | **Total** | **3652525** | **339** | **3652864** |  |
