# Supplementary material for: Uptake and cardiac events of COVID-19 vaccinations among Canadian youth and young adults
Source: PLOS Glob Public Health. 2024 Jul 31;4(7):e0003363. doi: 10.1371/journal.pgph.0003363 (PMC11290663; doi:10.1371/journal.pgph.0003363)
Supplement: S3 Table — (DOCX) [file pgph.0003363.s005.docx]

**S3 Table.** Odds of cardiac events by vaccine dose number from multivariate logistic regression models

|  | **Dose 1** | | | | **Dose 2** | | | | **Dose 3** | | | |
| --- | --- | --- | --- | --- | --- | --- | --- | --- | --- | --- | --- | --- |
| **Covariates** | **OR*** | **95% CI** | | **p-value** | **OR*** | **95% CI** | | **p-value** | **OR*** | **95% CI** | | **p-value** |
| *Vaccine Type* |  |  |  |  |  |  |  |  |  |  |  |  |
| Bivalent | N/A (Model cannot converge) | | | | N/A (Model cannot converge) | | | | 0.41 | 0.06 | 2.93 | 0.3708 |
| Original (reference) |  |  |  |  |  |  |  |  | 1.00 |  |  |  |
| *Population* |  |  |  |  |  |  |  |  |  |  |  |  |
| AAD | 0.92 | 0.58 | 1.47 | 0.7321 | 1.41 | 1.13 | 1.75 | 0.0023 | 1.13 | 0.67 | 1.91 | 0.6390 |
| Diabetes | 2.79 | 0.39 | 20.07 | 0.3090 | N/A (Model cannot converge) | | | | N/A (Model cannot converge) | | | |
| Diabetes with AAD† | 2.88 | 0.91 | 9.11 | 0.0724 | N/A (Model cannot converge) | | | | N/A (Model cannot converge) | | | |
| General Population (reference) | 1.00 |  |  |  | 1.00 |  |  |  | 1.00 |  |  |  |
| *Prior COVID Infection* |  |  |  |  |  |  |  |  |  |  |  |  |
| Yes | 0.76 | 0.40 | 1.45 | 0.4053 | 0.24 | 0.14 | 0.43 | <.0001 | 1.06 | 0.53 | 2.12 | 0.8727 |
| No (reference) | 1.00 |  |  |  | 1.00 |  |  |  | 1.00 |  |  |  |
| *Sex* |  |  |  |  |  |  |  |  |  |  |  |  |
| Female | 0.26 | 0.17 | 0.41 | <.0001 | 0.32 | 0.26 | 0.41 | <.0001 | 0.34 | 0.21 | 0.54 | <.0001 |
| Male (reference) | 1.00 |  |  |  | 1.00 |  |  |  | 1.00 |  |  |  |
| *Age* |  |  |  |  |  |  |  |  |  |  |  |  |
| 12-17 | 1.15 | 0.75 | 1.77 | 0.5163 | 1.32 | 1.07 | 1.65 | 0.0115 | 1.61 | 0.96 | 2.69 | 0.0708 |
| 18-35 (reference) | 1.00 |  |  |  | 1.00 |  |  |  | 1.00 |  |  |  |

* Also adjusted for location of residence, recency of immigration and income, instability, deprivation, dependency and ethnic diversity quintiles

†AAD stands for asthma and allergic diseases

OR – Odds Ratio

95% CI – 95% confidence interval
