# Supplementary material for: Uptake and cardiac events of COVID-19 vaccinations among Canadian youth and young adults
Source: PLOS Glob Public Health. 2024 Jul 31;4(7):e0003363. doi: 10.1371/journal.pgph.0003363 (PMC11290663; doi:10.1371/journal.pgph.0003363)
Supplement: S4 Table — (DOCX) [file pgph.0003363.s006.docx]

**S4 Table.** Odds of cardiac events by vaccine dose number from multivariable logistic regression models with acute cardiac events

|  | **Dose 1** | | | | **Dose 2** | | | | **Dose 3** | | | |
| --- | --- | --- | --- | --- | --- | --- | --- | --- | --- | --- | --- | --- |
| **Covariates** | **OR*** | **95% CI** | | **p-value** | **OR*** | **95% CI** | | **p-value** | **OR*** | **95% CI** | | **p-value** |
| *Vaccine Type* |  |  |  |  |  |  |  |  |  |  |  |  |
| Bivalent | 0.90 | 0.13 | 6.38 | 0.913 | N/A (Model could not converge) | | | | 0.83 | 0.41 | 1.67 | 0.593 |
| Original (reference) | 1.00 |  |  |  |  |  |  |  | 1.00 |  |  |  |
| *Population* |  |  |  |  |  |  |  |  |  |  |  |  |
| AAD | 1.45 | 1.23 | 1.71 | <.0001 | 1.27 | 1.10 | 1.46 | 0.001 | 1.17 | 0.89 | 1.53 | 0.261 |
| Diabetes | 2.61 | 1.24 | 5.51 | 0.012 | 2.03 | 0.97 | 4.29 | 0.062 | 1.61 | 0.40 | 6.50 | 0.502 |
| AAD with Diabetes | 2.82 | 1.80 | 4.41 | <.0001 | 1.62 | 0.98 | 2.65 | 0.058 | 2.30 | 1.14 | 4.65 | 0.021 |
| General Population (reference) | 1.00 |  |  |  | 1.00 |  |  |  | 1.00 |  |  |  |
| *Prior COVID Infection* |  |  |  |  |  |  |  |  |  |  |  |  |
| Yes | 0.60 | 0.47 | 0.78 | 0.0001 | 0.36 | 0.27 | 0.47 | <.0001 | 0.77 | 0.54 | 1.11 | 0.165 |
| No (reference) | 1.00 |  |  |  | 1.00 |  |  |  | 1.00 |  |  |  |
| *Sex* |  |  |  |  |  |  |  |  |  |  |  |  |
| Female | 0.87 | 0.76 | 1.00 | 0.056 | 0.71 | 0.63 | 0.80 | <.0001 | 0.94 | 0.76 | 1.16 | 0.564 |
| Male (reference) | 1.00 |  |  |  | 1.00 |  |  |  | 1.00 |  |  |  |
| *Age* |  |  |  |  |  |  |  |  |  |  |  |  |
| 12-17 | 0.80 | 0.67 | 0.96 | 0.018 | 1.22 | 1.06 | 1.39 | 0.004 | 1.30 | 0.98 | 1.73 | 0.065 |
| 18-35 (reference) | 1.00 |  |  |  | 1.00 |  |  |  | 1.00 |  |  |  |
| *Residence* |  |  |  |  |  |  |  |  |  |  |  |  |
| Urban | 0.95 | 0.69 | 1.29 | 0.728 | 0.86 | 0.67 | 1.10 | 0.219 | 1.25 | 0.74 | 2.10 | 0.414 |
| Rural (reference) | 1.00 |  |  |  | 1.00 |  |  |  | 1.00 |  |  |  |
| *Income Quintile* |  |  |  |  |  |  |  |  |  |  |  |  |
| 1 (Lowest) | 0.79 | 0.53 | 1.20 | 0.269 | 0.72 | 0.51 | 1.02 | 0.061 | 0.55 | 0.29 | 1.04 | 0.066 |
| 2 | 0.85 | 0.62 | 1.17 | 0.322 | 0.78 | 0.60 | 1.02 | 0.072 | 0.57 | 0.35 | 0.92 | 0.022 |
| 3 | 0.83 | 0.64 | 1.08 | 0.162 | 0.75 | 0.60 | 0.93 | 0.010 | 0.66 | 0.45 | 0.97 | 0.033 |
| 4 | 0.82 | 0.65 | 1.03 | 0.085 | 1.02 | 0.85 | 1.23 | 0.816 | 0.58 | 0.41 | 0.81 | 0.002 |
| 5 (Highest) | 1.00 |  |  |  | 1.00 |  |  |  | 1.00 |  |  |  |
| *Recent Immigrant* |  |  |  |  |  |  |  |  |  |  |  |  |
| Yes | 1.24 | 0.99 | 1.54 | 0.060 | 0.95 | 0.77 | 1.17 | 0.627 | 1.33 | 0.94 | 1.88 | 0.107 |
| No (reference) | 1.00 |  |  |  | 1.00 |  |  |  | 1.00 |  |  |  |

* Also adjusted for location of residence, recency of immigration and income, instability, deprivation, dependency and ethnic diversity quintiles

†AAD stands for asthma and allergic diseases

OR – Odds Ratio

95% CI – 95% confidence interval
