# Supplementary material for: Uptake and cardiac events of COVID-19 vaccinations among Canadian youth and young adults
Source: PLOS Glob Public Health. 2024 Jul 31;4(7):e0003363. doi: 10.1371/journal.pgph.0003363 (PMC11290663; doi:10.1371/journal.pgph.0003363)
Supplement: Attachment — Submitted filename: PGPH-D-24-00440_Response_to_Reviewers_TTO.docx [file pgph.0003363.s007.docx]

**RESPONSE TO REVIEWERS’ COMMENTS**

**Uptake and Cardiac Events of COVID-19 Vaccinations Among Canadian Youth and Young Adults**
[PGPH-D-24-00440]
*Please note that all line and page numbers cited correspond to the clean version of the revised manuscript.*

# COMMENTS FROM EDITOR:

1. **Please try to incorporate Appendix A and B into your supplement (or have another reference handy / or delete these sentences).**

Response: We have added the appendices to the supplement according to the editor’s request.

2. **The reviewer brings up a point about interpretation of risk ratios and odds ratios. I'm sure you just did (OR-1)*100% for the interpretation. I'm okay with that if you want to leave it in, but I also know that many readers don't learn that type of interpretation - so switching to X times higher odds using the OR directly might be better.**

Response: We have switched to using X times higher odds for greater clarity, as suggested.

# COMMENTS FROM REVIEWER #1:

1. **Line 11 and 217- the statistic of 39-127% higher odds of receiving booster doses. May i kindly request clarification of 127% odds of getting booster doses, does that mean more people (by a factor of 27% more) than those who got the vaccine ended up getting a booster dose or more (by 27%) people who got the covid 19 vaccine ended up getting more than 1 booster dose/or more than necessary booster doses.**

Response: These statistics intend to state that certain groups with comorbidities (AAD) have higher odds of seeking a booster dose compared to the general population. We have edited these lines for greater clarity.

2. **Line 15,34- was asthma and ‘allergic diseases’ the only co-morbidities included in the study? this comes from the antagonistic statement stated on line 65/66 which show an exclusion of Ontario residents with chronic conditions (possibly allergic conditions as well since the criteria of exclusion is not so clear) so a clear list of the ‘allergic’ conditions included would clarify this antagonism. This list is mentioned to be available as appendix A which as a reviewer i could not find.**

Response: Asthma and allergic diseases (allergic rhinitis or eczema) and diabetes were the common comorbidities we examined in this study. While allergic diseases were included in the original list (provided by the Government of Ontario) used for exclusions, we have removed them from the exclusion list since we intended to study them as comorbidities, as stated on line 68. The appendices have been added to the supplementary table file. For clarity, we have also included the table below.

**Appendix A.** Derived* Ontario’s COVID-19 Phase 2 vaccine guideline of at-risk health conditions.

|  | **Phase 2 Health Conditions** |
| --- | --- |
| **Solid organ transplant recipients** | Organ transplant recipients |
| **Hematopoietic stem cell transplant recipients** | Hematopoietic stem cell transplant recipients (bone marrow transplant) |
| **People with neurological diseases in which respiratory function may be compromised** | Neurological diseases that impact breathing (e.g., motor neuron disease, myasthenia gravis, multiple sclerosis) |
| **Hematological malignancy diagnosed < 1 year ago** | Hematological malignancy diagnosed within the last year |
| **Chronic kidney disease (with recent receipt of chronic dialysis)** | Kidney disease with estimated GFR <30 |
| **Current pregnancy** | Pregnancy |
| **High Risk Health Conditions** |  |
| **Other treatment causing immunosuppression** | Other treatments causing immunosuppression (for example, chemotherapy, immunity-weakening medications) |
| **Developmental disability** | Intellectual or developmental disabilities (for example, Down Syndrome) |
| **At risk** |  |
| **Immune deficiencies and autoimmune disorders** | Immune deficiencies and autoimmune disorders |
| **Stroke or cerebrovascular disease** | Stroke and cerebrovascular disease |
|  |  |
| **Dementia** | Dementia |
| **Liver disease** | Liver disease |
| **Chronic kidney disease** |  |
| **All other cancers** | All other cancers |
| **Respiratory diseases (COPD)** | Respiratory diseases, such as bronchitis, pneumonia, pleurisy |
| **Heart disease** | Heart disease |
| **Hypertension** | Hypertension with end organ damage |
| **Severe mental illness** | Diagnosed mental disorder |
| **Substance use disorder** | Substance use disorder |
| **Thalassemia** | Thalassemia |
| **Immunocompromising health conditions** | Immunocompromising health conditions Note: “Spleen” problems, such as “asplenia” and “Sickle cell disease” were combined with this characteristic. |
| **Pediatric conditions (12-17 years old only)** | |
| **Individuals with any pediatric complex chronic condition** |  |
| **Inflammatory bowel disease** |  |
| **Hemodynamically Significant Congenital heart disease (hs-CHD)** |  |
| **Bronchopulmonary dysplasia (BPD) or Congenital lung disease (CLD)** |  |
| **Cystic Fibrosis** |  |
| **Trisomy 21** |  |
| **Tracheostomy** |  |
| **Immunocompromising health conditions** |  |

This list and further information can be found at: <https://news.ontario.ca/en/backgrounder/60570/populations-eligible-for-phase-two-covid-19-vaccination>

* Asthma and diabetes were removed from the list since we studied them as comorbidities

3. **Line 67 and line 103- appendix A and appendix b are not available at the end of the document nor in the supplementary document provided as an .aspx file extension (on that note, the .aspx file extension is not easily opened on many devices, common pdfs and docx files could be considered).**

Response: We have added the appendices to the supplemental table file.

4. **Line 87- elaborate the acronym AAD as it is being used for the first time in this article**

Response: Line 87 is where we first define AAD as an acronym. We have edited the manuscript for greater clarity in accordance to the reviewer’s next comment.

5. **Line 88/89- are the examples given (allergic rhinitis and eczema) the only allergic disorders included or just examples because these 2 examples can easily be stated to be the only co-morbidities included**

Response: The reviewer is correct that these are the only two allergic diseases included. We have edited the line to simplify the language and increase the clarity of the AAD acronym.

# COMMENTS FROM REVIEWER #2:

1. **Abstract: Line 2 - Few studies have examined population data of the COVID-19 original and bivalent vaccine data on its uptake and potential side effects**

Response: We have edited the manuscript in accordance to the reviewer’s suggestion.

2. **Results: Line 147- Six hundred and sixteen (0.01%) individuals...**

Response: We have edited the manuscript in accordance to the reviewer’s suggestion and clarified that this percentage is taken form the total cohort of vaccinated and unvaccinated individuals.

3. **Line 149- Among those post-vaccinated individuals, 505(84.5%) had cardiac events within seven days and two -thirds (66.1%) of cardiac events occurring within two weeks post 2nd dose vaccination (Fig. 1)....**

Response: We have edited the manuscript in accordance to the reviewer’s suggestion.

4. **Table 1: The characteristics column could be such that 'The demographic factors is moved upwards then the N position as Total Population/age groups in same colored box**

Response: We have edited the manuscript in accordance to the reviewer’s suggestion.
